# Left Ventricular Thrombus Formation in a Young Female With a Severely Reduced Left Ventricular Ejection Fraction and a Recent Non-ST Segment Elevation-Acute Coronary Syndrome

**DOI:** 10.7759/cureus.17804

**Published:** 2021-09-07

**Authors:** Keshav R Patel, Mahmoud Kassir, Madhav Patel, Wesley Eichorn

**Affiliations:** 1 Internal Medicine, Western Michigan University Homer Stryker School of Medicine, Kalamazoo, USA; 2 Family and Community Medicine, Western Michigan University Homer Stryker School of Medicine, Kalamazoo, USA; 3 Neurology, Georgetown University School of Medicine, Washington, USA

**Keywords:** dilated cardiomyopathy, ventricular thrombus, nste-acs, hfref, amphetamine abuse, alcohol abuse

## Abstract

A 30-year-old female with a past medical history of heart failure with reduced ejection fraction (HFrEF of 20%), non-ST segment elevation-acute coronary syndrome (NSTE-ACS), and polysubstance abuse (heavy alcohol and methamphetamine use) was admitted for a heart failure exacerbation. Electrocardiogram and troponin levels were negative. Pro brain natriuretic peptide was elevated at 4,152 pg/mL. The patient was restarted on guideline-directed HFrEF therapy and continued to improve. Two days after presentation, the patient was transferred to the intensive care unit for severe alcohol withdrawal, requiring intravenous phenobarbital and dexmedetomidine. After her withdrawal symptoms resolved, she complained of right-sided weakness and stroke-like symptoms. Brain magnetic resonance imaging (MRI) and computed tomography (CT) were both negative. Echocardiography revealed an ejection fraction of 20% and a severely dilated left ventricle with a 2.1 x 1.2 cm apical density, suggestive of a thrombus, and the patient was started on apixaban. Echocardiography two months prior to this admission revealed an ejection fraction of 20%, but there was no evidence of a thrombus. Our patient had three major risk factors for left ventricular thrombus (LVT) formation: severely reduced left ventricular ejection fraction (LVEF), dilated cardiomyopathy (DCM), and a recent NSTE-ACS two months prior. This case highlights the importance of anticoagulation in patients at high risk for LVT formation and emphasizes the DCM may be seen in younger patients with heavy alcohol and amphetamine use.

## Introduction

Dilated cardiomyopathy (DCM), reduced left ventricular ejection fraction (LVEF), and acute coronary syndrome are common causes of ventricular thrombi [1]. According to Virchow’s Triad, thrombus formation can occur with blood stasis, blood vessel wall injury, and hypercoagulability. DCM, reduced LVEF, and acute coronary syndrome can result in ventricular akinesis or dyskinesis and therefore induce blood stasis. All of these conditions may also be accompanied by inflammation, blood vessel injury, and hypercoagulability. We present a unique case of polysubstance-induced DCM in a young female and a left ventricular thrombus (LVT) in the setting of DCM, severely reduced LVEF, and recent non-ST segment elevation-acute coronary syndrome (NSTE-ACS).

## Case presentation

A 30-year-old female with a past medical history of heart failure with a reduced ejection fraction (HFrEF) of 20%, recent NSTE-ACS, and polysubstance abuse (heavy alcohol and methamphetamine use) who presented with worsening dyspnea for two weeks. She also endorsed a weight gain of 20 pounds, chest tightness, and abdominal pain. Review of systems was negative for fevers, chills, cough, nausea, vomiting, or diarrhea.

The patient was afebrile with a heart rate of 117 beats/min, respiratory rate of 18 breaths/minute, blood pressure of 115/88 mmHg, and oxygen saturation of 100% on room air. On cardiovascular exam, the patient was tachycardic with +2 pulses in all four extremities. She also had an apical systolic murmur that was more pronounced while laying at 30 degrees. There was bilateral jugular venous distention with a positive hepatojugular reflux. On pulmonary exam, the patient had decreased breath sounds bilaterally.

Electrocardiogram revealed sinus tachycardia with diffuse lateral T wave inversions that were unchanged from previous tracings. A complete blood count was unremarkable. The complete metabolic panel revealed elevated liver tests: alkaline phosphatase of 178 U/L, aspartate transaminase of 92 U/L, and alanine transaminase of 94 U/L. Initial and repeat troponin levels were both normal and the patient was negative for COVID-19. Pro-brain natriuretic peptide was elevated at 4,152 pg/mL from a baseline of ~1,000 pg/mL. The urine drug screen was positive for marijuana and amphetamines.

**Figure 1:**
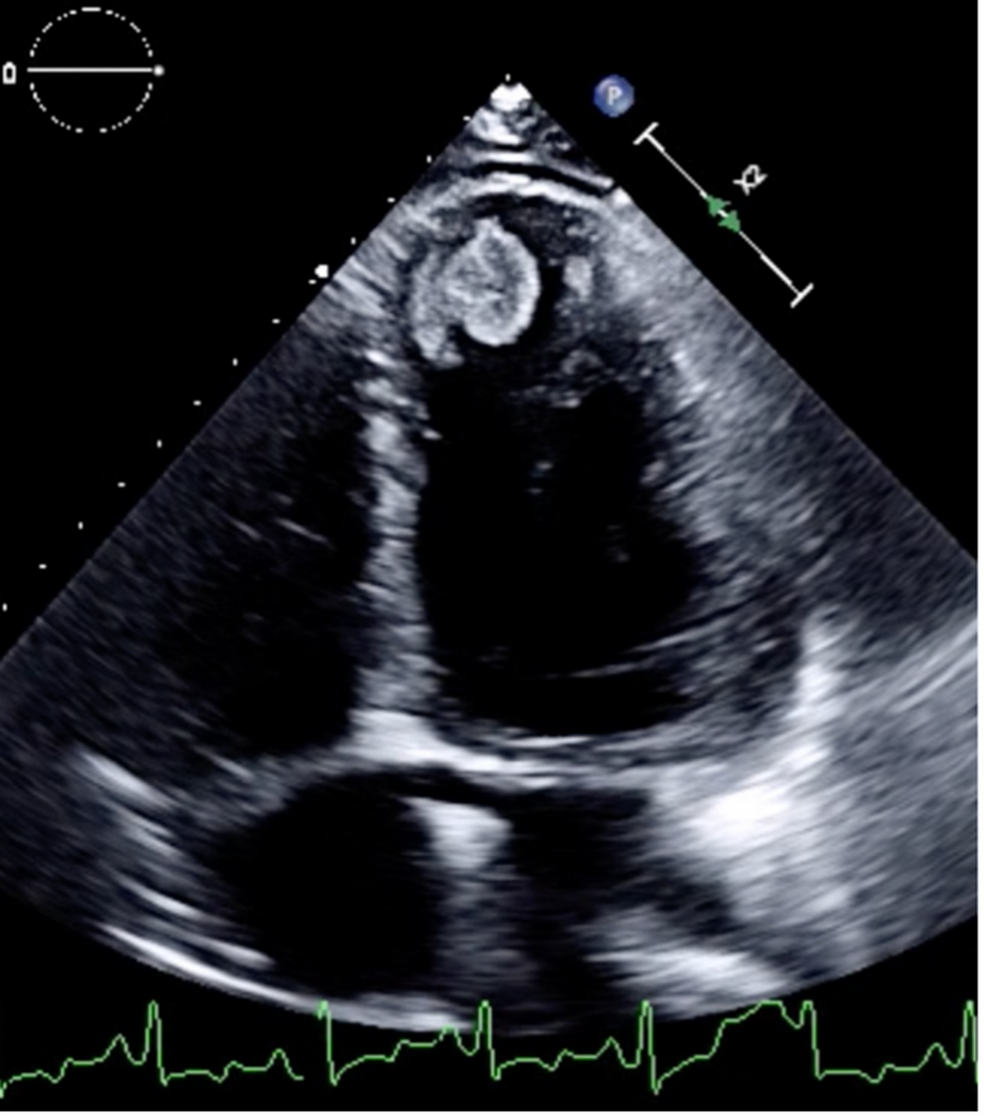
Apical Thrombus in Left Ventricle Echocardiography revealed a severely dilated left ventricle with a 2.1 x 1.2 cm apical density, suggestive of a thrombus and an ejection fraction of 20%.

Chest x-ray revealed cardiomegaly that was unchanged from previous imaging. Computed tomography (CT) angiography was negative for pulmonary embolism. Cardiology was consulted and determined that the patient did not require repeat echocardiography. The patient was non-compliant with guideline-directed medical therapy for HFrEF and was restarted on an ace inhibitor, diuretic, and beta-blocker during this hospitalization. She continued to improve, however, two days after presentation, she was transferred to the intensive care unit for severe alcohol withdrawal, requiring intravenous phenobarbital and dexmedetomidine. After her withdrawal had resolved, she complained of new-onset right-sided weakness in her upper extremity and the patient was worked up for a cerebrovascular accident (CVA). CT and magnetic resonance imaging (MRI) of the brain without contrast were negative. Echocardiography revealed an ejection fraction of 20% and a severely dilated left ventricle with a 2.1 x 1.2 cm apical density, suggestive of a thrombus (see Figure 1).

Echocardiography two months ago revealed an ejection fraction of 20% with a severely dilated left ventricle and no evidence of a thrombus. The patient was started on apixaban and discharged home. Of note, her right-sided weakness improved spontaneously during the hospitalization.

## Discussion

Historically, it was thought that alcohol-induced DCM required >80g of alcohol consumption for five years; however, a recent study showed that large amounts of alcohol over a short duration can cause an acute toxic myocardial dystrophy [2,3]. Amphetamine-induced DCM is not as common, but the incidence continues to increase [4]. Our patient used ~50-60 g of alcohol daily for the last five years. She also used amphetamines daily for the last few years. This case emphasizes the importance of early screening for high-risk alcohol and methamphetamine use and highlights that DCM may be seen in younger patients with these risk factors [2,3].

Our patient had three major risk factors for LVT formation: DCM, severely reduced LVEF, and an NSTE-ACS two months prior. Patients with an ejection fraction less than 40% or anteroapical wall motion dysfunction are considered high risk for LVT formation and should receive anticoagulation for at least three months [5]. Our patient was at high risk for LVT formation; however, she was non-compliant with home medications and therefore was not prescribed anticoagulation for prophylaxis. According to current guidelines, patients with a LVT should receive anticoagulation for at least three months [5,6] and our patient was discharged with apixaban.

The details of the patient's recent NSTE-ACS are unclear. The patient presented to an outside hospital and left against medical advice from the emergency department. During this current admission, cardiology determined that the patient did not need a cardiac catheterization. Of note, the patient had never undergone cardiac catheterization or stress testing in the past. Our patient was non-compliant with her lisinopril. It was previously thought that the use of lisinopril after an acute myocardial infarction (AMI) improved LV remodeling and therefore decreased the risk of thrombus, but a study found no difference in the rates of thrombus formation based on lisinopril use after AMI [7]. The location of the infarct also impacts the probability of thrombus formation. Patients with an ejection fraction less than 40% and an anterior AMI may have up to a 17.5% chance of thrombus formation [7]. Although we are unable to provide additional details on the patient's recent NSTE-ACS, it may be the primary driver of the LVT.

Early identification of a LVT is important because of an increased risk of systemic embolization, which occurs at an incidence of 11%, mostly within 3-4 months of thrombus detection [5]. There is limited data that suggests that the newer direct oral anticoagulants (DOACs) are good choices for LVT; however, guidelines still recommend warfarin [5,6]. Our patient had a significant history of medication non-compliance and leaving the hospital against medical advice, and therefore we chose apixaban. Since discharge, the patient has missed several appointments and has not been seen by her primary care physician or cardiologist.

Our patient did not require repeat echocardiography from a heart failure perspective; however, she exhibited stroke-like symptoms and had multiple risk factors for a LVT, including a low ejection fraction, severely dilated left ventricle, and recent NSTE-ACS. Brain CT and MRI were negative, and her stroke-like symptoms resolved during the hospitalization. This case highlights the importance of anticoagulation in patients at high risk for LVT.

## Conclusions

Ejection fraction less than 40%, DCM, and NSTE-ACS are important risk factors for LVT formation. According to current guidelines, patients at high risk for LVT formation should receive anticoagulation for at least three months. Our patient was at high risk for LVT formation but was not taking anticoagulation due to non-compliance. This case highlights the importance of anticoagulation in patients at high risk for LVT and emphasizes that DCM may be seen in younger individuals with heavy alcohol and methamphetamine use.
